# Cerebrospinal fluid Presenilin-1 increases at asymptomatic stage in genetically determined Alzheimer’s disease

**DOI:** 10.1186/s13024-016-0131-2

**Published:** 2016-09-29

**Authors:** Aitana Sogorb-Esteve, María-Salud García-Ayllón, Juan Fortea, Raquel Sánchez-Valle, Alberto Lleó, José-Luis Molinuevo, Javier Sáez-Valero

**Affiliations:** 1 Instituto de Neurociencias de Alicante, Universidad Miguel Hernández-CSIC, 03550, Av. Ramón y Cajal s/n, Sant Joan d’Alacant, E-03550 Spain; 2 Centro de Investigación Biomédica en Red sobre Enfermedades Neurodegenerativas (CIBERNED), Sant Joan d’Alacant & Barcelona, Spain; 3 Unidad de Investigación, Hospital General Universitario de Elche, FISABIO, 03203 Elche, Spain; 4 Memory Unit, Neurology Department, Hospital de la Santa Creu i Sant Pau, 08026 Barcelona, Spain; 5 Down Medical Center, Fundació Catalana Síndrome de Down, 08029 Barcelona, Spain; 6 Alzheimer’s Disease and Other Cognitive Disorders Unit, Neurology Service, Hospital Clinic; Institut d’Investigacions Biomèdiques Agust Pi i Sunyer, 08036 Barcelona, Spain

**Keywords:** Presenilin-1, Cerebrospinal fluid, Biomarker, Pre-symptomatic, Autosomal dominant Alzheimer’s disease, Down syndrome, Mild-cognitive impairment

## Abstract

**Background:**

Presenilin-1 (PS1), the active component of the intramembrane γ-secretase complex, can be detected as soluble heteromeric aggregates in cerebrospinal fluid (CSF). The aim of this study was to examine the different soluble PS1 complexes in the lumbar CSF (CSF-PS1) of individuals with Alzheimer’s disease (AD), particularly in both symptomatic and asymptomatic genetically determined AD, in order to evaluate their potential as early biomarkers.

**Methods:**

Western blotting, differential centrifugation and co-immunoprecipitation served to determine and characterize CSF-PS1 complexes. We also monitored the assembly of soluble PS1 into complexes in a cell model, and the participation of Aβ in the dynamics and robustness of the stable PS1 complexes.

**Results:**

There was an age-dependent increase in CSF-PS1 levels in cognitively normal controls, the different complexes represented in similar proportions. The total levels of CSF-PS1, and in particular the proportion of the stable 100–150 kDa complexes, increased in subjects with autosomal dominant AD that carried *PSEN1* mutations (eight symptomatic and six asymptomatic ADAD) and in Down syndrome individuals (ten demented and ten non-demented DS), compared with age-matched controls (*n* = 23), even prior to the appearance of symptoms of dementia. The proportion of stable CSF-PS1 complexes also increased in sporadic AD (*n* = 13) and mild-cognitive impaired subjects (*n* = 12), relative to age-matched controls (*n* = 17). Co-immunoprecipitation demonstrated the association of Aβ oligomers with soluble PS1 complexes, particularly the stable complexes.

**Conclusions:**

Our data suggest that CSF-PS1 complexes may be useful as an early biomarker for AD, reflecting the pathology at asymptomatic state.

**Electronic supplementary material:**

The online version of this article (doi:10.1186/s13024-016-0131-2) contains supplementary material, which is available to authorized users.

## Background

Alzheimer’s disease (AD) is a progressive neurodegenerative disorder that involves a gradual decline in memory and other cognitive functions, representing the most common cause of dementia in the elderly. Apart from the common late-onset forms of sporadic AD (sAD), rare mutations in the genes encoding the β-amyloid precursor protein (*APP*; chromosome 21q21), presenilin-1 (*PSEN1*; chromosome 14q24.3) and presenilin-2 (*PSEN2*; chromosome 1q31-q42) cause autosomal dominant AD (ADAD; also named as familial AD or FAD) [1]. ADAD exhibits similar phenotype as sAD but with an earlier clinical onset. The *APP* gene encodes a large type I transmembrane protein that upon proteolytic processing [2] can generate the β-amyloid peptide (Aβ), the major constituent of senile plaques and the triggering effector of AD. In the amyloidogenic pathway the Aβ peptide is generated by sequential cleavage of APP, starting with the cleavage of the large extracellular domain by the β-secretase cleaving enzyme (BACE1), which is followed by the successive action of γ-secretase at the membrane-spanning domain [3]. This γ-secretase is an intramembrane protease complex composed of presenilin-1 (PS1), nicastrin, APH1 (anterior pharynx-defective 1) and PEN2 (presenilin enhancer 2) [4]. PS1 is the catalytic subunit of the γ-secretase complex [5]. Duplications of APP and neighboring sequences are also linked to an early age of AD onset [6]. As such, Down’s syndrome (DS) is also associated with the development of AD since the APP gene lies on chromosome 21, and the extra copy leads to Aβ over-expression. Accordingly, most DS patients who live beyond the age of 40 years develop typical brain neuropathology AD and a significant proportion develop additional cognitive decline [7–9]. Thus, both these disease conditions, ADAD and DS, can be considered as early-onset forms of genetically determined AD [10].

Classic biomarkers, total and phospho-tau, as well as Aβ42, have shown diagnostic accuracy for incipient AD [11]. However total and phospho-tau also increased as a result of other neurological processes; while levels of the pathological Aβ42 species, which increased in the AD brain, resulted decreased in CSF due to increasing deposition, hindering the interpretation of changes in their soluble levels in early stages. Thus, there is still a need to identify additional early biomarkers. We recently demonstrated the presence of heteromeric PS1 complexes in human CSF (CSF-PS1) and serum, and that increases in the proportion of stable CSF-PS1 complexes served to discriminate sAD from non-disease controls [12]. PS1 is known to undergo endoproteolytic cleavage as part of its maturation, generating N- and C-terminal fragments (NTF and CTF) of about 29 and 20 kDa, respectively [13]. Both, the NTF and CTF of PS1 contain several transmembrane domains [14]; and our earlier data indicated that PS1 fragments might be highly unstable in CSF and serum, and that they spontaneously form complexes due to the large number of hydrophobic regions. Indeed, we demonstrated the presence of stable 100–150 kDa heteromeric complexes in CSF that contained the NTF and CTF of PS1 (maybe also involving other γ-secretase components), as well other large complexes. Some of these complexes were unstable under denaturing conditions and resolved as ~50 kDa heterodimers upon electrophoresis [12]. Moreover, an increase in the proportion of stable 100–150 kDa complexes appears to be a good marker to discriminate pathological AD samples from controls.

As such, we set out to further characterize these soluble PS1 complexes and the involvement of oligomeric Aβ in the formation of these complexes. We also evaluated the possibility that the proportions and nature of the CSF-PS1 complexes may vary during aging. The main interest was to investigate the levels of CSF-PS1 complexes in ADAD, sAD and DS, particularly in AD and DS subjects who had not yet developed dementia, including also mild-cognitive impaired (MCI) subjects. Thereby, we attempt to determine whether alterations to the levels of these complexes might reflect the pathological state at early, asymptomatic stages. Using a collection of well-characterized CSF samples from sAD PS1 complexes were also analyzed. Genetically determined AD offers unique opportunities to analyze diagnostic biomarkers at asymptomatic stages, particularly given that only in this group is a diagnosis guaranteed for the early comparison of biomarkers.

## Methods

### Patients

Lumbar CSF samples were obtained from ADAD subject that were all carriers of *PSEN1* mutations and who were part of the Genetic Counseling Program (PICOGEN) at the Hospital Clínic, Barcelona [15]. This group included 14 subjects carrying PSEN1 mutations (including six asymptomatic mutation carriers), and eight age-matched non-mutation carriers from the same families (younger non-disease controls: yNC). The clinical and CSF data of some of these patients has been reported previously [16, 17]. We also included lumbar CSF samples from 10 DS subjects with Alzheimer’s type dementia (dDS) and 10 DS subjects without signs of memory decline (ndDS) obtained at the Hospital Sant Pau, Barcelona, along with 15 additional age-matched yNC obtained from both hospitals. In addition, 15 patients with dementia due to sAD, 12 subjects with MCI and 17 age-matched elderly controls (eNC) were also obtained from the Hospital Sant Pau, Barcelona. See Table 1 for details of clinical and demographic data. All AD patients fulfilled the 2011 NIA-AA criteria for dementia or MCI due to AD [18, 19], while discrimination between the dDS subjects and those without dementia was assessed using the modified Cued Recall Test and the CAMDEX-DS battery [20, 21]. All the control subjects had no history or symptoms of neurological or psychiatric disorders, or memory complaints. This study was approved by the ethics committee at the Miguel Hernandez University and it was carried out in accordance with the Declaration of Helsinki.

**Table 1:** Clinical, demographic data and classic CSF biomarker levels

Group	Age (years)	n (Gender)	MMSE score	CSF Aβ42 (pg/mL)	CSF T-tau (pg/mL)	CSF P-tau (pg/mL)
yNC {yNC member of the same ADAD families}	45 ± 2 [25–60] {37 ± 3 [25–47]}	n = 23 (15 F/8 M) {6 F/2 M}	29 ± 1 [25–30] {29 ± 1 [28–30]}	809 ± 44 {791 ± 84}	207 ± 15 {231 ± 29}	43 ± 3 {45 ± 11}
syADAD	45 ± 3 [31–59]	n = 8 (5 F/3 M)	21 ± 2* [11–28]	300 ± 54*	899 ± 186*	164 ± 60*
psADAD	36 ± 3 [24–41]	n = 6 (4 F/2 M)	30 ± 1 [29, 30]	1120 ± 252	222 ± 26	49 ± 5
dDS	55 ± 2 [43–61]	n = 10 (5 F/5 M)	ND	411 ± 24*	788 ± 125*^,a^	106 ± 14*^,a^
ndDS	43 ± 2 [33–49]	n = 10 (5 F/5 M)	ND	570 ± 51*	232 ± 53	45 ± 8
eNC	67 ± 1 [61–80]	n = 17 (11 F/6 M)	29 ± 1 [26–30]	753 ± 30	197 ± 12	42 ± 2
sAD	68 ± 2 [54–83]	n = 13 (9 F/4 M)	20 ± 1** [18–24]	351 ± 17**	833 ± 87**	135 ± 18**
MCI due to AD	66 ± 1 [61–72]	n = 12 (5 F/7 M	26 ± 1** [20–30]	422 ± 31	618 ± 66**	81 ± 8**

In the yNC group (younger controls), the values for the control subgroup of non-mutation carriers from the same families as the carriers of *PSEN1* mutations are also indicated; the rest of cases correspond to subject without family history of ADAD. The *PSEN1* mutations included in this study from syADAD cases (“symptomatic” autosomal dominant AD subjects) corresponded to 3 carriers of L286P, and one of I439S, S169P, L173F, L235R and L282R. Those psADAD subjects (pre-symptomatic subjects carrying mutations in *PSEN1*) were 3 carriers of M139T, and one of I439S, R220G and K239N. Patients with (dDS) or without (ndDS) signs of clinical dementia were also compared with yNC; sporadic AD (sAD) and mild-cognitive impaired (MCI) subjects were compared with elderly controls (eNC). Levels of Aβ42, T-tau and P-tau were determined by ELISA; the intra-assay coefficient of variability (CV) was below 5 % and inter-assay CV below 15 % for all the classical AD biomarkers, in agreement with previous reports [36]. The number of samples “n” for female (F) and male (M) subjects is indicated. The data represent the means ± SEM, and for age and MMSE (Minimental State Examination), the range of values is also indicated. *Significantly different (*p* <0.05) from the yNC group, ^a^and from the ndDS group; **Significantly different (*p* <0.05) from the eNC group

### PS1 over-expressing cells silencing by siRNA

CHO cells (400,000 cells/well) were grown in DMEM® (Gibco) containing 10 % Fetal Bovine Serum (Gibco) and 1 % Penicillin/Streptomycin (Sigma-Aldrich), and they were transfected with a construct encoding full-length PS1 (2 μg cDNA) [22] or with the pcDNA3 expression plasmid alone (Invitrogen), using Lipofectamine 2000® (Invitrogen). To reduce the PS1 gene expression we used CHO cells stably over expressing wild-type human PS1 and APP (CHO-PS1/APP) [23]. CHO-PS1/APP cells (350,000 cells/well) were grown in DMEM® containing 10 % Fetal Bovine Serum, 0,1 % Puromicin (Sigma-Aldrich) and 0,2 % G418 disulfate salt (Sigma-Aldrich), were transfected with BLOCK iT™ Alexa Fluor® Red Fluorescent Oligo (Invitrogen) as control, or siRNA (50 nM) targeting human PS1 (Santa Cruz Biotechnology, INC). Without removing the cell media, 24 h after the first transfection cells were transfected with the same siRNA (30 nM) and incubated for an additional 18 h.

### Western blotting and immunoprecipitation

Although the denaturation temperature prior to electrophoresis has not been standardized, we found that high temperature sample preparation for electrophoresis (98 °C) produced an overall loss of CSF-PS1 immunoreactivity [24]. Hence, all analyses of in this study PS1 avoided freeze-thaw cycles (samples were aliquoted), and denaturation prior to electrophoresis was conducted at 50 °C.

Samples (30 μL for CSF) were resolved by sodium dodecyl sulfate-polyacrylamide gel electrophoresis (SDS-PAGE) under reducing conditions. The proteins were then transferred to nitrocellulose membranes (Schleicher and Schuell Bioscience GmbH) that were probed with PS1 antibodies directed against the N-terminal amino acids 1–20 (antibody 98/1) [24]. GAPDH (Abcam) served as a loading control for cellular extracts. Membranes were incubated with the corresponding horseradish peroxidase conjugated secondary antibody and the immunoreactive signal was detected in a Luminescent Image Analyzer LAS-1000 Plus (FUJIFILM) using SuperSignal West Dura Extended Duration Substrate (Thermo Scientific). A control CSF sample was used to normalize the immunoreactive signal, and for semi-quantitative studies the intensity of the immunoreactive bands was measured by densitometry using Science Lab Image Gauge v 4.0 software provided by FUJIFILM. Aβ peptides in CSF immunoprecipitates (see below) were resolved by 16 % Tris-tricine SDS-PAGE and detected with the 6E10 antibody (Covance Research).

For immunoprecipitation, samples were precleared for 2 h at 4 °C by incubation with protein A-Sepharose (Sigma-Aldrich). Immunoprecipitations were performed at 4 °C by incubating 150 μL of CSF or cell media, overnight with the primary PS1 C-terminal antibody 00/2 (raised against residues 301–317) [23] previously coupled to protein A-Sepharose using Dimethyl pimelimidate dihydrochloride (Sigma-Aldrich Co). Precipitated proteins were washed with PBS and eluted with 0.1 M glycine buffer at pH 2.5. After pH neutralization, supernatants were denatured in Laemmli sample buffer at 50 °C for 15 min and subjected to SDS-PAGE. The membranes were then probed with anti-PS1 (98/1) and anti-Aβ (6E10) antibodies.

### Sucrose gradients

PS1 complexes were analyzed by ultracentrifugation for 4 h at 4 °C on a continuous sucrose density gradient (5–20 %) at 250,000 × *g*. CSF aliquots (65 μL) were carefully loaded onto the top of the gradient containing 2 mL of 0.15 M NaCl, 50 mM MgCl_2_ and 0.5 % Brij 97 in 50 mM Tris-HCl (pH 7.4). After centrifugation, ~14 fractions were collected gently from the top of the tubes. Enzyme markers of known sedimentation coefficient, β-galactosidase, catalase and alkaline phosphatase were used in the gradients to determine the approximate sedimentation coefficients. The sucrose fractions containing highly stable and unstable PS1 complexes were pooled separately, dialyzed against Tris buffer and concentrated by ultrafiltration (Amicon Ultra 10,000 MWCO, Millipore Corporation, Bedford, MA). The PS1 complexes were then immunoprecipitated with anti-PS1 00/2 as described.

### Measurement of T-tau, P-tau and Aβ42 by ELISA

The CSF levels of total tau (T-tau), phosphorylated tau (P-tau) and Aβ1-42 (Aβ42) were determined using specific enzyme-linked immunosorbent assays (ELISA: Fujirebio Europe, Ghent, Belgium).

### Statistical analysis

All data were analyzed using SigmaStat (Version 3.5; Systac Software Inc.), applying a one-way analysis of variance or a Kruskal-Wallis test when the hypothesis of equality of sample variances was rejected. Pairwise group comparisons were then sustained using Student *t* test (two-tailed) or Mann-Whitney U test, and the exact *p* values determined. The results are presented as the means ± SEM, and correlations between the variables were assessed by linear regression analyses, with *p* values <0.05 considered statistically significant.

## Results

### The increase in CSF-PS1 with age

**Fig. 1:**
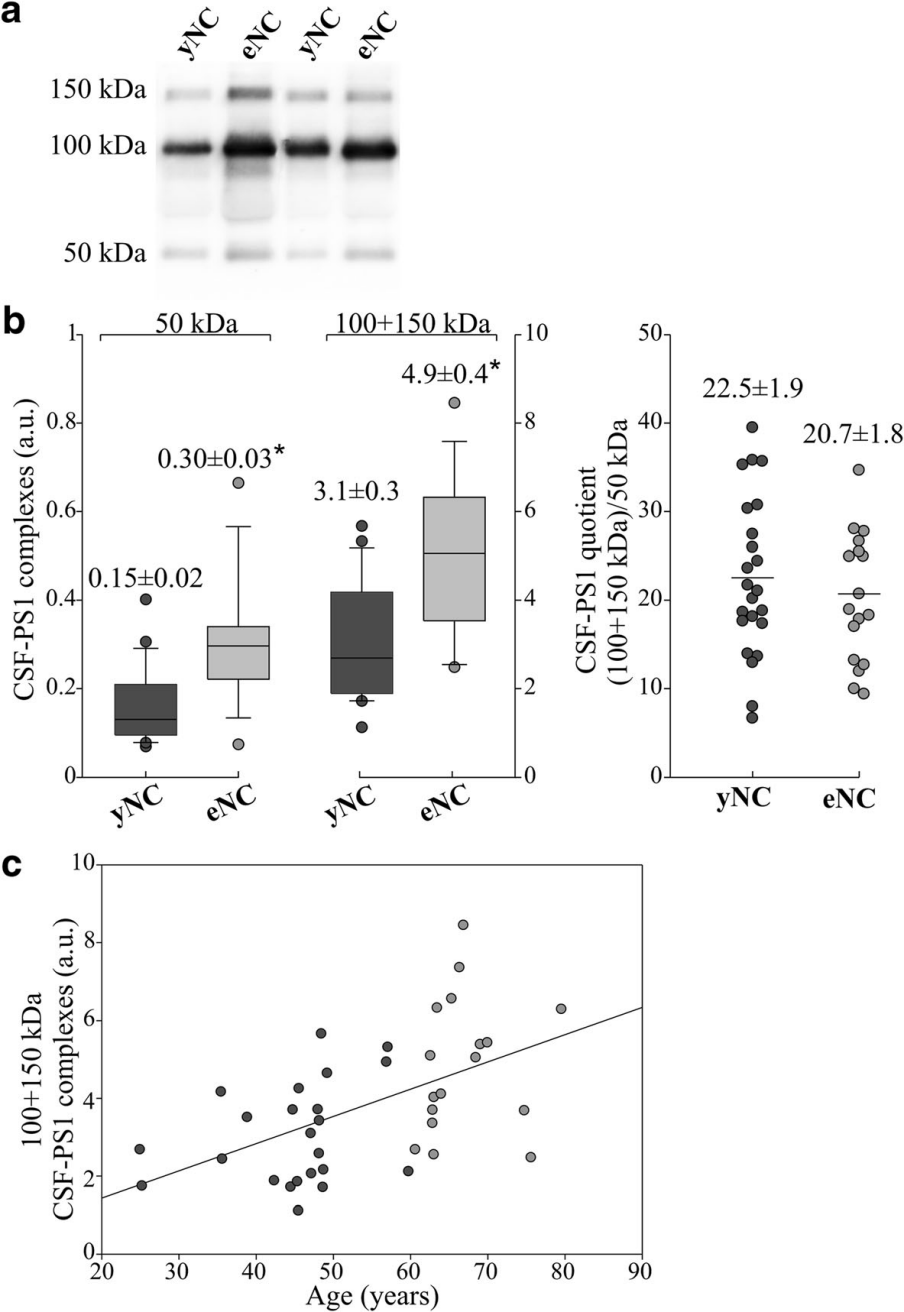
Characterization of the CSF-PS1 complexes in younger and elderly NC subjects, and their correlation with age. **a** Representative Western blots of human CSF samples from non-demented control (NC) subjects arbitrarily categorized as young (yNC; ≤60 years; *n* = 23) and elderly (eNC; >60 years; *n* = 17), and probed with an anti-NTF-PS1 antibody. **b** Densitometric quantification of the major 100 and 150 kDa CSF-PS1 complexes (the sum of the 100 + 150 kDa CSF-PS1 bands) and the quotient derived from the immunoreactivity for the 100 and 150 kDa bands relative to that for the minor 50 kDa band in each sample [(100 + 150 kDa)/50 kDa]. The data represent the means ± SEM and they were compared using a paired Students *t* test: *p <0.001. **c** Correlation between the levels of the 100 + 150 kDa CSF-PS1 complexes with age

Since the main aim of the present study was to determine the changes in CSF-PS1 associated with ADAD and DS, and given that both ADAD and DS exhibits earlier clinical onset, we first assessed whether the amount and nature of the soluble PS1 complexes varies with age. The PS1 complexes in samples from control subjects (NC) from 25 to 80 years-of-age were detected with the 98/1 antibody, which predominantly recognized complexes of approximately 100 and 150 kDa, together with a less abundant 50 kDa band (Fig. 1a). The identity of these bands as complexes involving NTF- and CTF-PS1 was demonstrated in a previous study [12]. This soluble 50 kDa PS1 band may represent a NTF and CTF-PS1 aggregate, as the holoprotein had a mass of ~43 kDa and it differs in its electrophoretic migration [12]. PS1-NTF monomers are not detectable in human CSF samples. Since ADAD starts prior to 60 years of age [1], we sub-grouped young and elderly NC below and above this threshold. The sum of the immunoreactivity for the major 100 and 150 kDa PS1 complexes was significantly higher (~58 %) in the elderly NC (eNC; *n* = 18) than in the young NC samples (yNC; *n* = 19; *p* <0.001: Fig. 1b). No differences were found between values obtained from the two center of sample collection. In all the NC samples, the major 100 and 150 kDa PS1 complexes were positively correlated with age (*r* = 0.54; *p* <0.001: Fig. 1c). Therefore, this age-dependent increase in PS1 complexes must be taken into account when comparing the different pathological groups with non-disease subjects, defining appropriate age-matched controls.

We also attempted to assess potential differences in the class of the PS1 complexes in the NC sub-groups based on the direct analysis of the Western blots. As such, we defined the (100 + 150 kDa)/50 kDa quotient for each sample. No change was observed in the (100 + 150 kDa)/50 kDa quotient evaluated in CSF from yNC and eNC subjects (Fig. 1b).

### Higher PS1 levels in symptomatic and asymptomatic ADAD

**Fig. 2:**
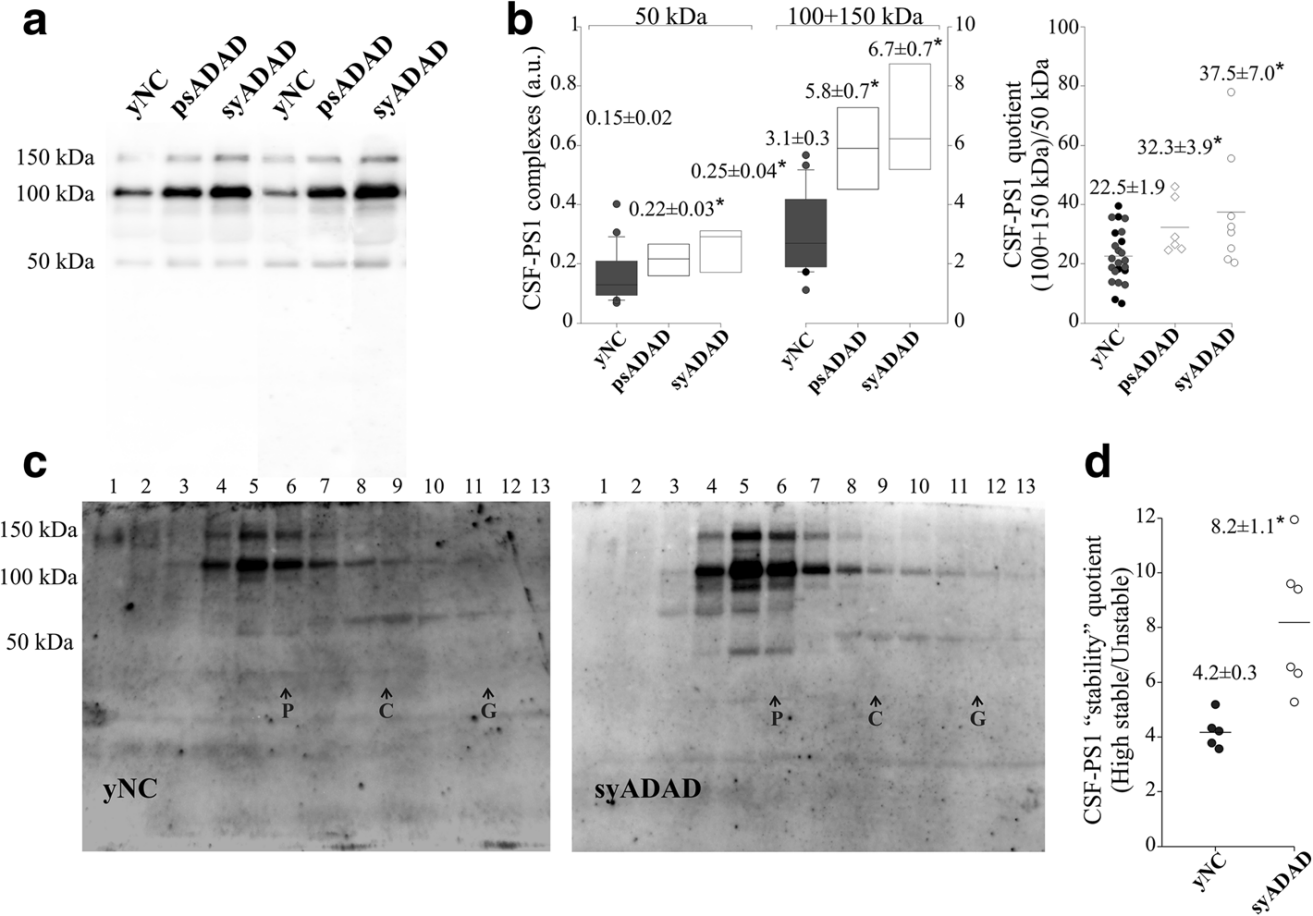
The increase in the CSF-PS1 complexes in ADAD. **a** Representative blot of the PS1 complexes in the CSF samples from eight symptomatic ADAD (syADAD), six presymptomatic mutation carriers (psADAD) and 23 younger NC controls (yNC), eight of which were from the same families a the ADAD subjects but that did not carry mutations (black symbol; see also Table 1). **b** Densitometric quantification of the accumulative immunoreactivity from the sum of the higher molecular mass PS1 complex (100 + 150 kDa). A quotient was calculated for each sample defined as the sum of (100 + 150 kDa) immunoreactivity relative to the 50 kDa immunoreactivity: (100 + 150 kDa/50 kDa). **c** Six syADAD and five yNC samples were fractionated on 5–20 % sucrose density gradients to further characterize the PS1 complexes. The fractions (collected from the top of each tube) were immunoblotted under denaturing conditions and probed for PS1, as in (**a**). β-Galactosidase (G, 16.0S; ~540 kDa), catalase (C, 11.4S; ~232 kDa) and alkaline phosphatase (P, 6.1S; ~140–160 kDa) were used as internal markers. Representative blots are shown. **d** The “stability” quotient was defined as the sum of the stable immunoreactive bands that sediment close to alkaline phosphatase (~140–160 kDa; fractions 2–7), mainly the 100 and 150 kDa bands, relative to the large unstable complexes that sediment closer to catalase (~232 kDa; fractions 8–12), and resolve mainly as 50 kDa immunoreactive bands in Western blots. The data are the means ± SEM: *Significantly different (*p* <0.005) from the yNC group as assessed by the Student *t* or Mann-Whitney U tests

To assess whether the amount of CSF-PS1 is altered in ADAD, the levels in the age-matched yNC group were compared with those in the CSF from symptomatic (syADAD) and asymptomatic (pre-symptomatic: psADAD) subjects carrying mutations in *PSEN1* in Western blots (see Table 1 and Fig. 2a). Stronger immunoreactivity for the 100 and 150 kDa complexes was evident in syADAD (~119 %; *p <*0.001) and in psADAD (~87 %; *p* <0.001) subjects compared to the yNC, with no differences between the two pathological groups (Fig. 2b). Indeed, the levels in these AD subjects were significantly higher than in the yNC sub-group, composed by non-mutation carriers from the same ADAD families (*p <*0.001). The previously defined quotient of CSF-PS1 complexes (see above) also discriminated between the yNC and the two ADAD groups, both individually (*p =* 0.007 for syADAD; *p* = 0.027 for psADAD) or when considered as a unique pathological group (*p =* 0.007). Thus, a higher proportion of 100 + 150 kDa CSF-PS1 complexes appears to be associated with ADAD even at pre-symptomatic stages (Fig. 2b).

PS1 complexes can be also characterized by gradient ultracentrifugation [24], followed by Western blotting under denaturing conditions, which served to illustrate the existence of different CSF-PS1 complexes [12]. When, CSF-PS1 complexes from yNC and syADAD subjects were characterized by sedimentation analysis on sucrose density gradients (Fig. 2c), 100–150 kDa PS1 complexes were identified close to the alkaline phosphatase marker (~140–160 kDa), along with larger complexes that sedimented in regions closer to the catalase marker (~232 kDa). These latter complexes were unstable and resolved as 50 kDa peptides by SDS-PAGE/Western blot analysis (Fig. 2c). In good agreement with results with the CSF-PS1 complex quotient obtained for direct Western blot analysis, samples separated by ultracentrifugation revealed higher abundance of the highly stable 100–150 kDa PS1 complexes in the syADAD samples than in the yNC samples, more so than the complexes of the 50 kDa fragments that sedimented in the denser fractions. This difference was clearly evident with the determination of a refined quotient, the “stability” quotient, reflecting the differences between the highly stable complexes (the 100–150 kDa heterodimers that sediment close to the internal marker of similar molecular mass) and the unstable complexes (the 50 kDa complexes that sediment closer to catalase), this quotient allowing us to discriminate syADAD (*p* = 0.004) from yNC samples (Fig. 2d).

### Highly stable CSF-PS1 complexes are elevated in sAD and MCI

**Fig. 3:**
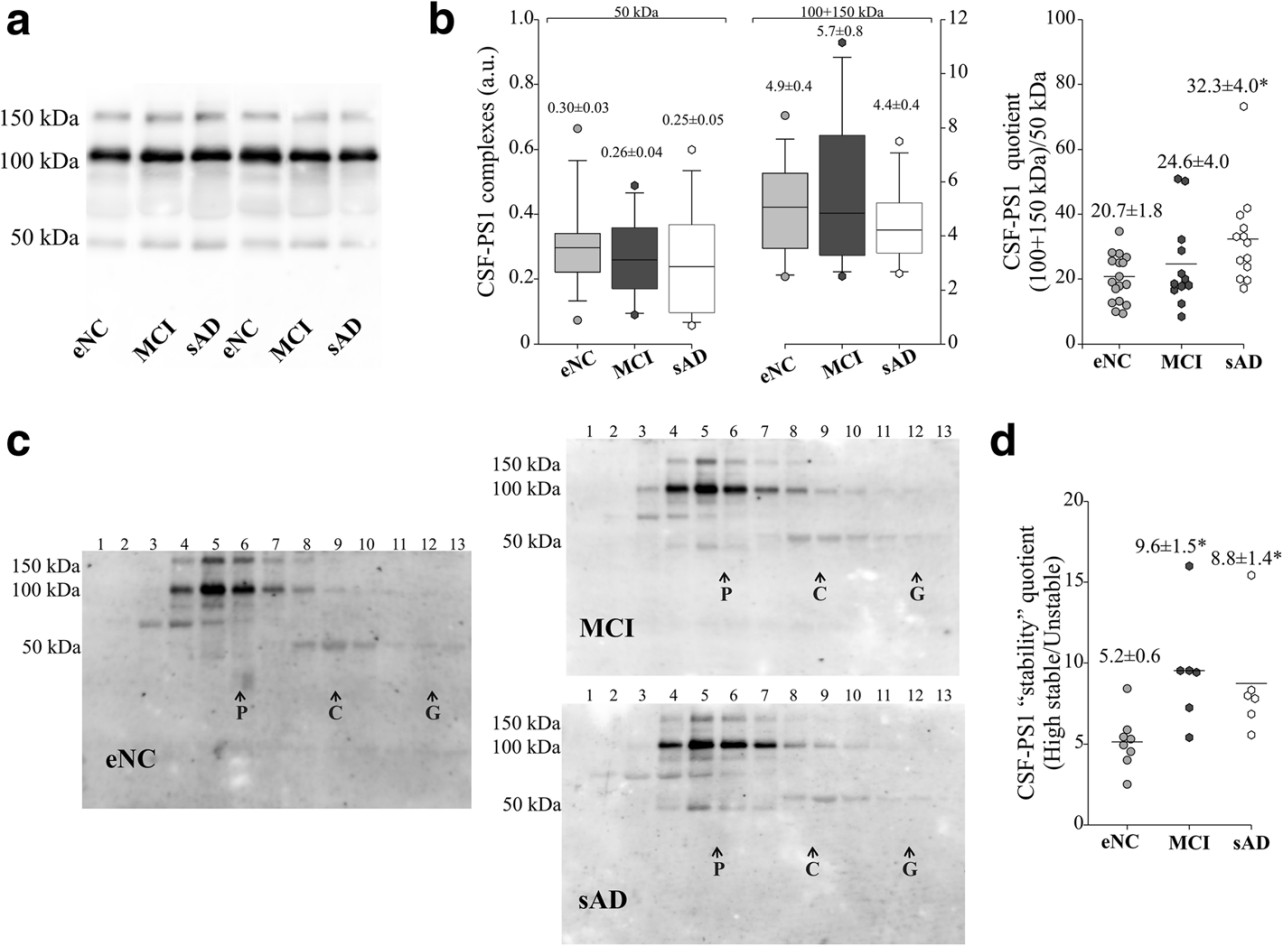
Increase in the stable PS1 complexes in AD and MCI CSF. **a** Representative blot and (**b**) densitometric quantification of the accumulative immunoreactivity from the sum of stable higher molecular mass PS1 complexes (100 + 150 kDa) in CSF samples from 13 sAD, 12 MCI and 17 age-matched eNC subjects. A quotient calculated as the sum of (100 + 150 kDa) immunoreactivity relative to the 50 kDa immunoreactivity: (100 + 150 kDa/50 kDa) is also shown. **c** Six AD and MCI individuals, and 8 eNC subjects were fractionated on 5–20 % sucrose density gradients, and probed with the PS1 antibody under denaturing conditions. The internal markers were β-galactosidase (G), catalase (C) and alkaline phosphatase (P), as in Fig. 3. **d** The values for the “stability” quotient reflecting the highly stable complexes (100 + 150 kDa immunoreactive bands sedimenting in fractions 2–7) relative to the unstable complexes (50 kDa immunoreactive bands sedimenting in fractions 8–12) is also shown. *p <0.05, **p <0.01 as assessed by the Student *t* or Mann-Whitney U tests

In sAD no notable differences in total PS1 were observed between patients with dementia due to sAD, MCI due to AD, or age-matched eNC subjects (Fig. 3a, b). However, the highly stable PS1 complexes were again more abundant in probable sAD cases compared to elderly eNCs when the CSF-PS1 complexes quotient was calculated (*p* = 0.006; Fig. 3b). Sucrose density centrifugation profiles (Fig. 3c) and the subsequent estimation of the “stability” quotient confirmed the greater abundance of highly stable PS1 complexes in sAD compared to eNC (*p* = 0.02; Fig. 3d), as well as indicating that the highly stable complexes were particularly increased in MCI subjects (*p* = 0.008; Fig. 3d).

### Higher PS1 levels in demented and non-demented DS

**Fig. 4:**
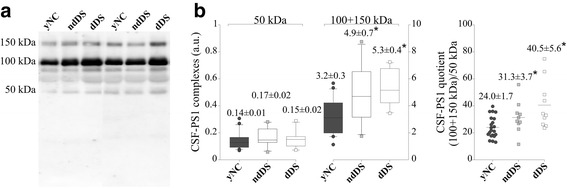
An increase in PS1 stable complexes in DS CSF. **a** Representative blot of PS1 complexes in CSF from 10 DS subjects with dementia of the Alzheimer’s type (dDS), 10 DS without any sign of memory decline (ndDS) and 23 yNC. **b** Densitometric quantification of the accumulated immunoreactivity from the sum of the higher molecular mass PS1 complex (100 + 150 kDa), and the quotient of the (100 + 150 kDa) immunoreactivity relative to the 50 kDa immunoreactivity (100 + 150 kDa/50 kDa). The means ± SEM are shown: *p <0.005, **p <0.005, Student *t* test

DS is considered a pre-symptomatic AD [10]. To assess whether an increase in the CSF-PS1 complexes is also associated with DS, we analyzed CSF samples from DS patients with (dDS) or without (ndDS) signs of clinical dementia, comparing these to age-matched yNC (Fig. 4a). The cumulative immunoreactivity of the major 100 and 150 kDa bands was significantly higher in both dDS (*p <*0.001) and ndDS (*p* = 0.007) CSF than in that from yNC subjects (Fig. 4b). Remarkably, the CSF-PS1 complexes quotient also revealed consistent changes in the proportion of the different complexes for both dDS (*p <*0.001) and ndDS subjects (*p* = 0.04) relative to yNC (Fig. 4b).

### The formation of stable CSF-PS1 complexes is favored by β-amyloid

Although PS1 clearly forms native complexes in CSF, there is little knowledge about the dynamics of soluble PS1 fragment assembly into heteromeric complexes. Thus, we monitored the assembly of soluble PS1 into complexes in a cell model, CHO cells over-expressing wild-type human PS1. An increase in the 29 kDa NTF of PS1 in extracts from CHO cells transfected with human PS1 corroborated that these cells over-expressed the protein (Additional file 1: Figure S1A). Immunoblotting of the cell-conditioned medium revealed predominant bands of approximately 100 and 150 kDa, and a weaker ~70 kDa band. The amounts of these soluble PS1 complexes increased in conditioned media from CHO cells transfected with PS1 (Additional file 1: Figure S1A). CHO cells stably transfected with PS1 and APP showed similar soluble PS1 complexes with additional 50 kDa band and monomeric NTF (Additional file 1: Figure S1A). To ascertain the identity of the soluble PS1 complexes in the cellular model, we reduced PS1 expression in CHO cells stably over expressing wild-type human PS1 with siRNA PS1. Cells transfected with siRNA PS1 displayed decrease in cellular PS1-NTF, but also in soluble PS1 complexes identified in cell media (Additional file 1: Figure S1A).

We also analyzed the soluble PS1 complexes in the conditioned medium of PS1-transfected CHO cells and CHO cells over-expressing PS1 and APP, using sucrose-density gradient fractionation followed by Western blotting under denaturing conditions (Additional file 1: Figure S1B). The majority of the soluble PS1 in the CHO cell-conditioned medium accumulated close to the alkaline phosphatase marker (~140–160 kDa) and resolved as 70 kDa complexes after denaturation, with only faint bands at 100 kDa. However, some 29 kDa monomeric PS1 was also evident, probably released from the complexes (Additional file 1: Figure S1B). By contrast, in the medium of CHO cells over-expressing PS1 and APP there was virtually no 29 kDa NTF immunoreactivity, indicating that in the context of β-amyloid over-expression, most of the soluble PS1 is stably incorporated into complexes (Additional file 1: Figure S1B).

We further tested the possible interaction between soluble PS1 complexes and Aβ. PS1 was immunoprecipitated from the medium of CHO cells over-expressing PS1 and APP with the 00/2 antibody that recognizes the PS1 CTF. Immunoprecipitation of heteromeric PS1 complexes was confirmed in Western blots probed with the anti-N-terminal 98/1 antibody (Additional file 1: Figure S1C). Considerable amounts of Aβ oligomers were also detected in these immunoprecipitates by the 6E10 antibody (Additional file 1: Figure S1C), while no immunoreactivity was resolved by a C-terminal APP antibody (not shown); indicating that oligomers of Aβ, but not C-terminal fragments, interact with the soluble PS1 complexes.

**Fig. 5:**
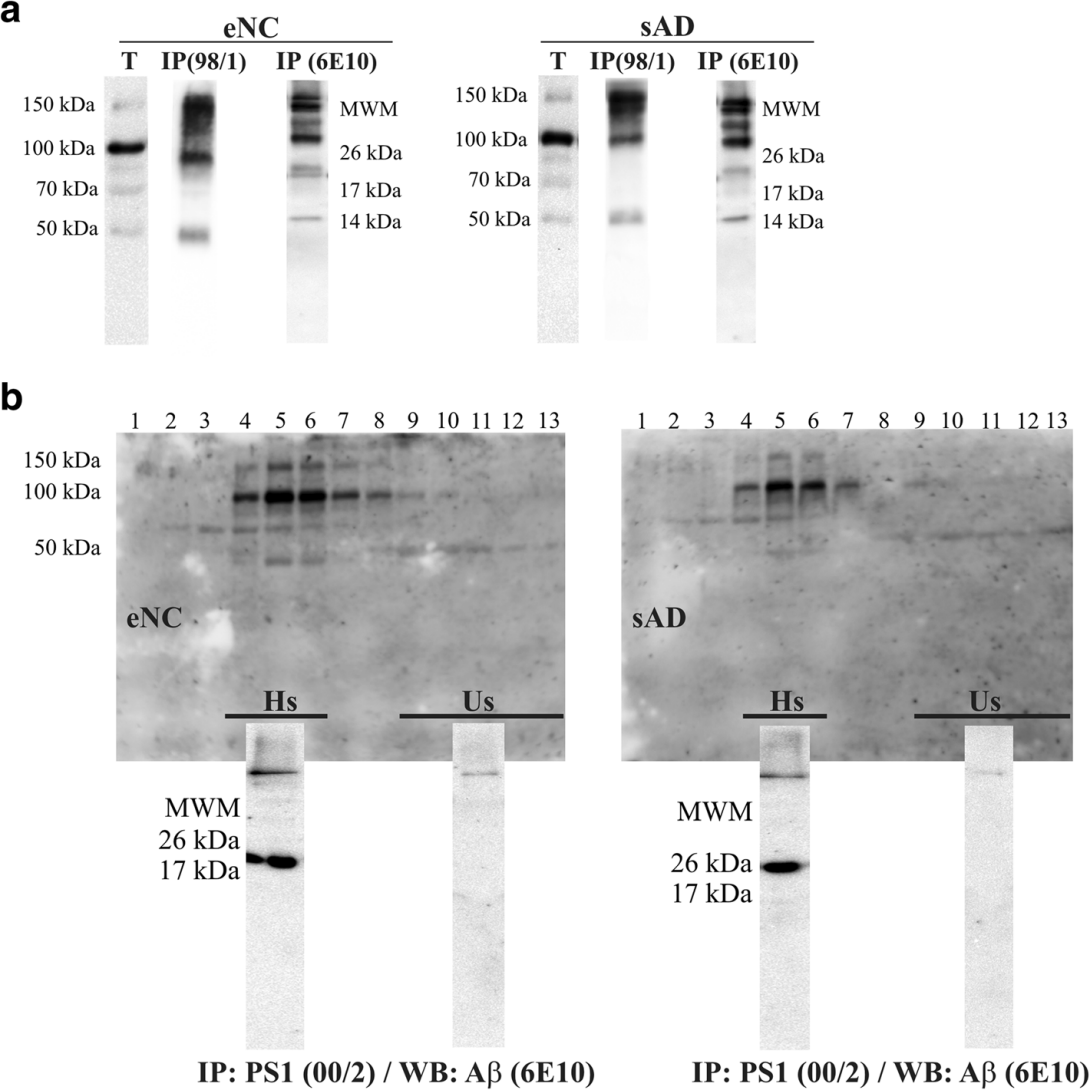
Aβ oligomers are present in highly stable CSF-PS1 complexes. **a** CSF samples from eNC and sAD subjects were precleared with protein A-Sepharose (T: total), and then immunoprecipitated with the anti CTF-PS1 00/2 antibody. The precipitated proteins (IP) were probed in immunoblots with the antibody indicated (98/1 for NTF-PS1 and 6E10 for Aβ). Note that oligomeric Aβ species co-immunoprecipitate and interact with CSF-PS1 complexes in both eNC and sAD. None immunoreactivity was resolved in negative controls incubated with beads in the absence of antibody (not shown). **b** CSF-PS1 complexes were fractionated by sucrose gradient centrifugation, and the fractions containing highly stable (Hs) or unstable (Us) PS1 complexes were pooled, dialyzed and concentrated by ultrafiltration. Representative sedimentation profiles illustrate the fractions selected for peak isolation. The enriched CSF-PS1 complexes were then immunoprecipitated with the 00/2 antibody and assayed in immunoblots probed with the 6E10 antibody against Aβ (insert). Representative blots reveal that Aβ oligomers are mainly present in peak fractions containing highly stable PS1 complexes (illustrative examples from two different experiments)

To confirm that Aβ oligomers favors the formation of stable PS1 complexes in human CSF we examined the Aβ peptides in PS1 complexes immunoprecipitated from CSF samples from sAD and eNC subjects. Again, CSF samples immunoprecipitated with 00/2 antibody were probed in immunoblots with the 98/1 and 6E10 antibodies (Fig. 5a), demonstrating that Aβ oligomers co-immunoprecipitated with heteromeric PS1 complexes from both eNC and sAD CSF samples. We further tested the involvement of Aβ on the formation of the highly stable PS1 complexes. After CSF-PS1 complexes were fractioned by sucrose density gradients and the peak fractions of the highly stable and unstable complexes were isolated, they were immunoprecipitated with the 00/2 antibody (Fig. 5b). Aβ oligomers were clearly present in the fractions rich in stable 100–150 kDa complexes from both eNC and sAD samples, whereas virtually no Aβ immunoreactivity was detected in the pooled fractions of 50 kDa PS1 complexes (Fig. 5b). Hence, oligomers of Aβ appear to mainly associate with the highly stable PS1 complexes.

## Discussion

The detection of soluble PS1 in CSF and serum [12] was a somewhat unexpected finding, particularly since PS1 is a multi-pass transmembrane protein with several hydrophobic regions [14]. Indeed, the presence of soluble PS1 has been reported in the medium of primary neurons [25] and confirmed in human serum [26]. Here, we corroborated the existence of different PS1 complexes in human CSF and we revealed their potential utility as a biomarker for AD. Like many membrane proteins, PS1 has a tendency to aggregate under non-native conditions [27, 28]. Thus, CSF-PS1 complexes probably represent non-specific aggregates of PS1 NTF and CTF distinct to the active γ-secretase membrane-complexes [12].

How PS1 complexes become soluble and appear in the CSF is yet to be determined. However, it appears that Aβ oligomers can probably contribute to the formation of stable CSF-PS1 complexes which are particularly abundant in AD. Indeed, it is remarkable that when we follow the formation of PS1 complexes in the cell-conditioned media, the co-expression of APP and PS1 favored the accumulation of complexes and not soluble monomeric PS1 is existent. We were able to pull down oligomeric Aβ species by PS1 immunoprecipitation from the medium, as well as from human CSF, in which Aβ oligomers are mainly associated to the highly stable PS1 complexes. Aβ peptides are chemically “sticky”, gradually building up into fibrils and aggregates; although the mechanism of how can Aβ stabilize CSF-PS1 is yet to be determined. Also in this context, levels of soluble Aβ peptide assessed by ELISA determinations appear consistently decreased in AD CSF [11]. The possibility that some amounts of Aβ participate within stable protein complexes in CSF, resulting underestimated by conventional ELISA protocols, may deserve consideration.

In CSF samples from NC subjects we observe an age-related increase in the total amount of PS1, while the relative proportion of the different complexes remains unaltered. No changes were observed comparing NC samples from different center of sample collection or gender. However, the relative proportion of stable PS1 complexes does appear to increase in the AD condition.

We propose that the most significant phenomenon related to the potential use of CSF-PS1 to discriminate the pathological state is the change in the proportion of PS1 complexes, rather than the estimates of the total PS1 levels. Accordingly, we focused our analysis on the highly stable 100–150 kDa PS1 complexes in CSF. The highly stable CSF-PS1 complexes co-exist with unstable complexes, sedimenting after differential centrifugation in regions closer to 200–250 kDa, but mainly resolved as 50 kDa components by reducing SDS-PAGE. We found that a quotient of PS1 complexes can discriminate all pathological groups from age-matched controls. We suggest that these quotients reflect differences in the properties of the PS1 complexes formed under pathological conditions. Screening large numbers of samples by sucrose gradient ultracentrifugation is difficult. As a reliable alternative, we addressed the discrimination of samples using a complementary parameter, a quotient of CSF-PS1 complexes calculated directly from Western blot analysis [(100 + 150 kDa)/50 kDa], thereby simplifying the analysis. This alternative quotient is useful to discriminate ADAD and DS subjects from age-matched yNC, as well as sAD from eNC. In our analysis, this quotient of PS1 complexes only failed to adequately discriminate MCI subjects, maybe indicating a lack of sensibility with respect to the evaluation of the complexes after separation by ultracentrifugation in sucrose density gradients. The inherent uncertainty in clinical diagnosis may also account for these differences, particularly for MCI group in which some subjects maybe remained MCI stable or develop to other dementias.

Anyhow, large overlap is observed between groups when assessment of the relative amount of CSF-PS1 complexes is estimated by a quotient obtained directly from Western blot analysis, without fractioning by ultracentrifugation. It will be necessary to replicate these finding using other techniques, such as ELISA specific for stable CSF-PS1 complexes, to evaluate their true potential as biomarkers.

Interestingly, altered levels of CSF-PS1 are detectable in both symptomatic and asymptomatic ADAD subjects. Similarly, alterations to CSF-PS1 levels occur in DS subjects with and without dementia. The analysis of CSF samples from DS subjects is of particular interest since it is well known that almost all adults with DS over 40 years of age display AD neuropathology [29, 30], although the prevalence of dementia in these individuals varies considerably [31–34]. Thus, there is no association between the age of onset of AD neuropathology in DS subjects and the appearance of clinical dementia [35], and we cannot predict the number of ndDS that will develop future cognitive impairment. In the view of the consistent changes in CSF-PS1 in ndDS we assume that this biomarker is more related to the brain pathological status than the occurrence of dementia and cognitive decline.

## Conclusions

In conclusion, our present findings demonstrate that CSF-PS1 complexes are altered in genetically determined AD, as well as in sAD. Together, our results indicate that the increase in stable PS1 complexes in CSF is an early phenomenon associated to AD pathology and may constitute an asymptomatic biomarker.

## Additional file

Additional file 1: Figure S1. Aβ affects the dynamics and stability of the soluble PS1 complexes. (**A**) CHO cell were transfected with human PS1 or with the pcDNA3 expression plasmid as a control (Ø). CHO cells stably over-expressing PS1 and APP were also transfected with PS1 siRNA. PS1 in the cell extracts and soluble PS1 complexes in the medium were assayed in Western blots using a NTF-PS1 antibody (equivalent amounts of protein of the cell extracts and equal volumes of medium were loaded in each lane). GAPDH served as a loading control for cellular extracts. (**B**) Soluble PS1 complexes from the medium conditioned by CHO cells transfected with PS1 (CHO-PS1), or CHO cells over-expressing PS1 and APP (CHO-PS1/APP), characterized by ultracentrifugation on 5–20 % sucrose density gradients. Fractions were collected from the top of each tube and they were analyzed by SDS-PAGE under denaturing conditions. Enzymes of known sedimentation coefficient were used as internal markers: β-galactosidase (G, 16.0S; molecular mass ~540 kDa), catalase (C, 11.4S; ~232 kDa) and alkaline phosphatase (P, 6.1S; ~140–160 kDa). Note that both PS1 complexes and 29 kDa monomers were identified in CHO-PS1 cells, while the 29 kDa monomers are mostly absent from the CHO-PS1/APP cells. (**C**) Cell medium conditioned by CHO-PS1/APP cells was precleared with protein A-Sepharose and the soluble PS1 complexes were immunoprecipitated with the anti-PS1 antibody 00/2 raised against the CTF. The immunoprecipitated proteins (IP) were probed with the 98/1 antibody against the NTF of PS1 and the 6E10 antibody against Aβ (T: total). The PS1 antibody confirms the immunoprecipitation of heteromeric complexes of PS1 and the 6E10 confirms that these PS1 complexes contain or interact with small Aβ oligomers. Illustrative examples from three different experiments are shown. (TIF 2441 kb)

## Abbreviations

AD: Alzheimer’s disease
ADAD: Autosomal dominant AD
APP: β-amyloid precursor protein
Aβ: β-amyloid peptide
CSF: Cerebrospinal fluid
CTF: C-terminal fragment of PS1
dDS: DS patients with dementia
DS: Down syndrome
eNC: Elderly NC
MCI: Mild-cognitive impairment
NC: Normal control subjects
ndDS: DS patients without dementia
NTF: N-terminal fragment of PS1
PS1: Presenilin-1
psADAD: Pre-symptomatic ADAD
P-tau: Phosphorylated tau
sAD: Sporadic Alzheimer’s disease
syADAD: Symptomatic ADAD
T-tau: Total tau
yNC: Young NC samples
