# Supplementary figures and images for: Cerebrospinal fluid Presenilin-1 increases at asymptomatic stage in genetically determined Alzheimer’s disease
Source: Mol Neurodegener. 2016 Sep 29;11:66. doi: 10.1186/s13024-016-0131-2 (PMC5043603; doi:10.1186/s13024-016-0131-2)

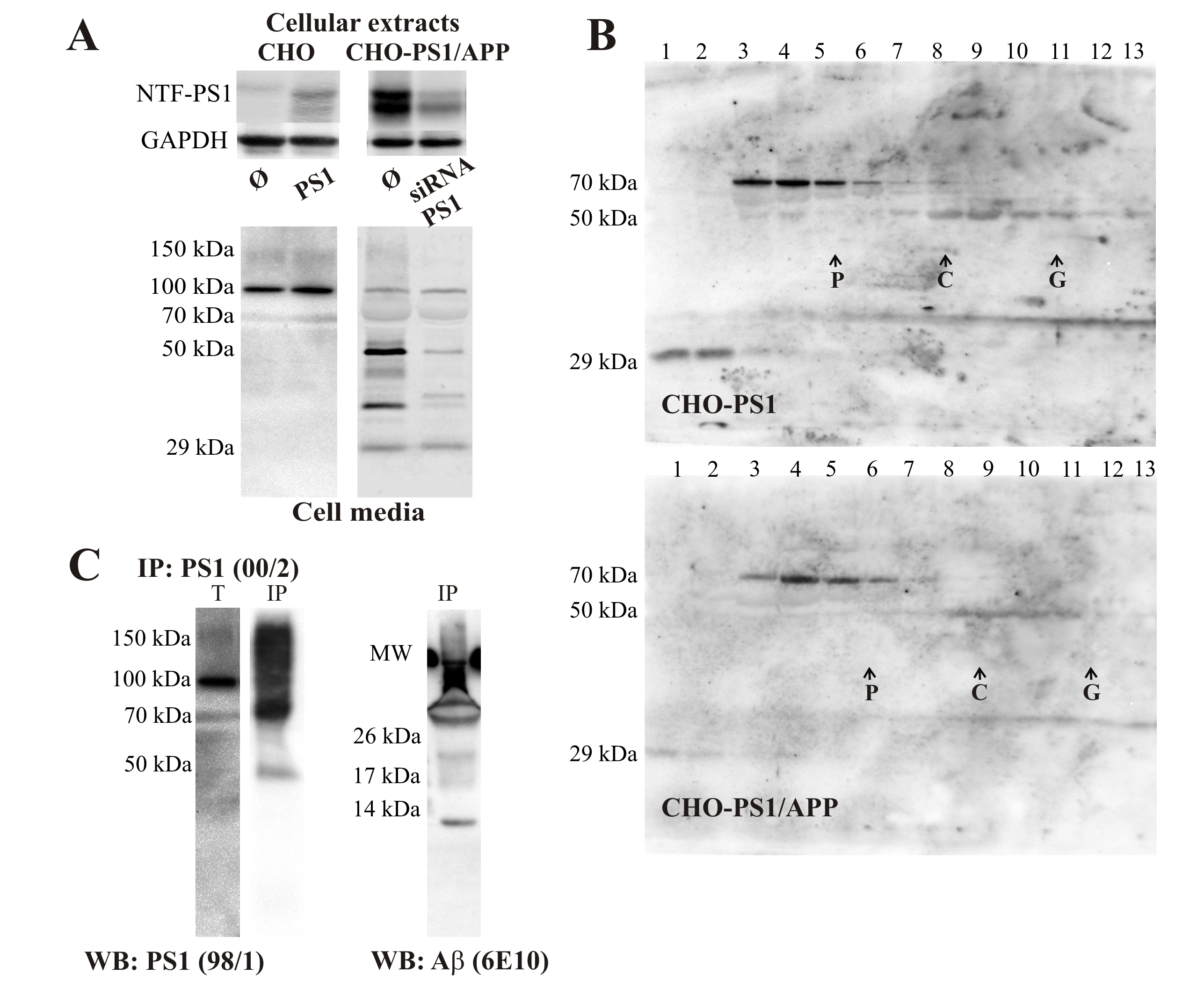

Supplement: Additional file 1: Figure S1. — Aβ affects the dynamics and stability of the soluble PS1 complexes. (A) CHO cell were transfected with human PS1 or with the pcDNA3 expression plasmid as a control (Ø). CHO cells stably over-expressing PS1 and APP were also transfected with PS1 siRNA. PS1 in the cell extracts and soluble PS1 complexes in the medium were assayed in Western blots using a NTF-PS1 antibody (equivalent amounts of protein of the cell extracts and equal volumes of medium were loaded in each lane). GAPDH served as a loading control for cellular extracts. (B) Soluble PS1 complexes from the medium conditioned by CHO cells transfected with PS1 (CHO-PS1), or CHO cells over-expressing PS1 and APP (CHO-PS1/APP), characterized by ultracentrifugation on 5–20 % sucrose density gradients. Fractions were collected from the top of each tube and they were analyzed by SDS-PAGE under denaturing conditions. Enzymes of known sedimentation coefficient were used as internal markers: β-galactosidase (G, 16.0S; molecular mass ~540 kDa), catalase (C, 11.4S; ~232 kDa) and alkaline phosphatase (P, 6.1S; ~140–160 kDa). Note that both PS1 complexes and 29 kDa monomers were identified in CHO-PS1 cells, while the 29 kDa monomers are mostly absent from the CHO-PS1/APP cells. (C) Cell medium conditioned by CHO-PS1/APP cells was precleared with protein A-Sepharose and the soluble PS1 complexes were immunoprecipitated with the anti-PS1 antibody 00/2 raised against the CTF. The immunoprecipitated proteins (IP) were probed with the 98/1 antibody against the NTF of PS1 and the 6E10 antibody against Aβ (T: total). The PS1 antibody confirms the immunoprecipitation of heteromeric complexes of PS1 and the 6E10 confirms that these PS1 complexes contain or interact with small Aβ oligomers. Illustrative examples from three different experiments are shown. (TIF 2441 kb) [file 13024_2016_131_MOESM1_ESM.tif]
